# Chinese Herbal Medicine for Acute Mountain Sickness: A Systematic Review of Randomized Controlled Trials

**DOI:** 10.1155/2013/732562

**Published:** 2013-12-22

**Authors:** Jie Wang, Xingjiang Xiong, Yanwei Xing, Zhen Liu, Wenrui Jiang, Junyi Huang, Bo Feng

**Affiliations:** ^1^Department of Cardiology, Guang'anmen Hospital, China Academy of Chinese Medical Sciences, No. 5 Beixiange, Xicheng District, Beijing 100053, China; ^2^Department of Gastroenterology, Guang'anmen Hospital, China Academy of Chinese Medical Sciences, Beijing 100053, China

## Abstract

*Objectives*. We aimed to assess the current clinical evidence of Chinese herbal medicine for AMS. *Methods*. Seven electronic databases were searched until January 2013. We included randomized clinical trials testing Chinese herbal medicine against placebo, no drugs, Western drugs, or a combination of routine treatment drugs against routine treatment drugs. Study selection, data extraction, quality assessment, and data analyses were conducted according to Cochrane standards. *Results*. Nine randomized trials were included. The methodological quality of the included trials was evaluated as low. Two trials compared prescriptions of Chinese formula used alone with Western drugs. A meta-analysis showed a beneficial effect in decreasing the score of AMS (MD: −2.23 [−3.98, −0.49], *P* = 0.01). Only one trial compared prescriptions of Chinese formula used alone with no drugs. A meta-analysis showed a significant beneficial effect in decreasing the score of AMS (MD: −6.00 [−6.45, −5.55], *P* < 0.00001). Four trials compared Chinese formula used alone with placebo. A meta-analysis also showed a significant beneficial effect in decreasing the score of AMS (MD: −1.10 [−1.64, −0.55], *P* < 0.0001). Two trials compared the combination of Chinese formula plus routine treatment drugs with routine treatment drugs. A meta-analysis showed a beneficial effect in decreasing the score of AMS (MD: −5.99 [−11.11, −0.86], *P* = 0.02). *Conclusions*. No firm conclusion on the effectiveness and safety of Chinese herbal medicine for AMS can be made. More rigorous high-quality trials are required to generate a high level of evidence and to confirm the results.

## 1. Introduction

High-altitude areas are defined as areas at altitudes equal to or greater than 2700 m above mean sea level [1]. Each year, millions of people ascend to altitudes between 2000 and 4000 m. The Golmud-Lhasa railroad constructed in Tibet, China, has 30,000–50,000 workers at high altitudes, including many who work at more than 4000 m. Acute mountain sickness (AMS) is an unpleasant syndrome that has been described in many persons who ascend rapidly to high altitude [2, 3]. This syndrome is characterized by symptoms of headache, nausea or vomiting, dyspnea, fatigue, poor appetite, dizziness, and difficulty in sleeping [4, 5]. Onset of AMS occurs within 24 h of hypoxic exposure, often within the first few hours. The incidence of AMS was first reported in 1976. Hackett and colleagues found that 53% of 278 unacclimatized hikers suffered from AMS at an altitude of 4.173 km in the Himalayas of Nepal [6]. An American study reported an incidence of AMS of 25% at 1.89–2.91 km [7]. A Chinese study on construction workers of the Qinghai-Tibet railroad (altitudes up to 5000 m) showed that the overall incidence of AMS upon first-time exposure was 51% [8].

The pathophysiology of AMS is not well understood. The essential factor responsible for this condition is hypoxemia. Hypoxia during ascent to high altitude is responsible for an increase in pulmonary arterial pressure [9, 10]. In 1991, the Lake Louise questionnaire scoring system was developed by the International Hypoxia Symposium aiming to standardize the assessment of AMS [11, 12]. Currently, most patients who have mild to moderate AMS do not need specific treatment, and symptoms abate in 2-3 days. Severe AMS can be treated by several therapies (acetazolamide, dexamethasone, oxygen, and descent), which are efficacious in hastening recovery [13]. A minority of people develop more severe manifestations of AMS (e.g., high-altitude cerebral edema) and need to be transported to lower altitudes or receive medical treatment [14].

Currently, with increasing application of complementary and alternative medicine worldwide, traditional Chinese medicine (TCM) has become more popular and drawn more attention [15–19]. TCM has formed a particular way on diagnosis and treatment of disease [20–23]. Approximately 50% of US residents use some form of alternative medicine [24]. Recent research has shown that complementary and alternative medicine (especially integrative medicine) contributes to the therapy of AMS [25]. The efficacy of TCM for treating AMS has been suggested by a large number of published case series and randomized trials, although some trials have demonstrated negative results [26]. At present, Chinese formulas used alone or combined with Western drugs are widely used as an alternative and effective method for the treatment of AMS in China. Clinical studies of Chinese formulas have reported their clinical effect, including case reports and case series to controlled observational studies and randomized clinical trials.

However, there is no critically appraised evidence, such as systematic reviews or meta-analyses, on the potential benefits of TCM on AMS. Therefore, it is difficult to justify clinical use and recommendation of various Chinese formulas. This study aimed to assess the current clinical evidence of Chinese formulas for AMS.

## 2. Materials and Methods

### 2.1. Database and Search Strategies

We selected all of the clinical trials on Chinese decoctions or traditional medicine monomers used for treating AMS in the Chinese National Knowledge Infrastructure (CNKI), Chinese Biomedical Literature Database (CBM), Chinese Scientific Journal Database (VIP), Wanfang data, PubMed, Embase, and the Cochrane Central Register of Controlled Trials in the Cochrane Library (January 2013). We also searched reference lists of retrieved papers. Databases in Chinese were searched to retrieve the maximum possible number of trials of Chinese formulas or traditional medicine monomers for AMS because they are mainly used in China. The following search terms were used individually or combined: “acute mountain sickness,” “acute high altitude disease,” “herb,” “herbal medicine,” “Chinese herbal medicine,” “Chinese drug,” “compound prescription,” “traditional Chinese medicine,” “traditional Chinese medical,” “decoction,” “Chinese formula,” “Chinese medicine monomer,” “controlled clinical trial,” and “clinical trial.”

### 2.2. Inclusion Criteria

All of the parallel, randomized controlled trials (RCTs) of all Chinese decoctions or traditional medicine monomers compared with Western drugs, placebo, no drugs, or routine treatment in patients with AMS were included. RCTs on Chinese decoctions combined with Western drugs compared with a control group were also included. There were no restrictions on population characteristics, language, or publication type. The main outcome measure was the score of AMS. Duplicated publications reporting the same groups of participants were excluded.

### 2.3. Data Extraction and Quality Assessment

Two authors conducted the literature search, study selection, and data extraction independently (Feng and Xiong). The extracted data included authors, title of the study, year of publication, study design, number of participants, details of intervention (herbs were included), details of control interventions, outcomes, intervention durations, and main findings. Disagreement was resolved by discussion and consensus was reached through a third party (Wang).

We assessed the methodological quality of trials by criteria of the Cochrane Handbook for Systematic Review of Interventions, version 5.1.0 [27]. Assessment involved six criteria, including random sequence generation (selection bias), allocation concealment (selection bias), blinding of participants and personnel (performance bias), blinding of outcome assessment (detection bias), incomplete outcome data (attrition bias), and selective reporting (reporting bias).

### 2.4. Data Synthesis

Revman 5.1 software provided by the Cochrane Collaboration was used for data analyses. Dichotomous data are expressed as the relative risk and continuous outcomes as the weighted mean difference, both with 95% confidence intervals (CIs). Meta-analysis was performed if the intervention, control, and outcome were the same or similar. Statistical heterogeneity was presented as significant when *I* square (*I* ^2^) was >50% or *P* < 0.1. In the absence of significant heterogeneity, we pooled data using a fixed-effects model (*I* ^2^ < 50%), and, otherwise, we used a random-effects model (*I* ^2^ > 50%) [28].

## 3. Results

### 3.1. Description of Included Trials

**Figure 1:**
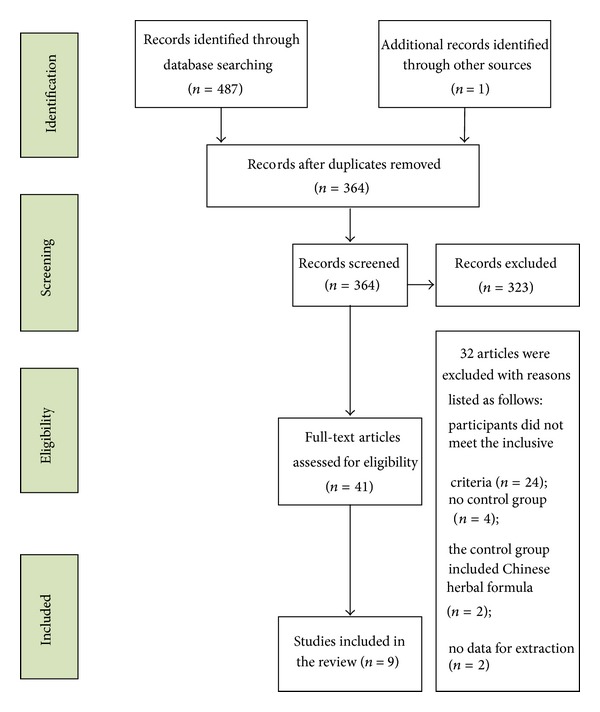
Study selection process.

After a primary search of five databases, 364 trials were screened out from electronic and manual searches (Figure 1), and the majority were excluded because of obvious ineligibility, which included irrelevant titles and abstracts. After reading the titles and abstracts, 364 trials were excluded because of duplicated publication. The rest of the 323 trials were case reports, case series, traditional reviews, or not rigorously designed RCTs. After the above selection, only 41 studies were obtained. Twenty-four of the remaining 41 articles were excluded based on the inclusion criteria. In the rest of the 17 trials, four of them had no control group, two had a control group using a Chinese herbal formula as the control group, and two trials had no data for extraction. Finally, nine RCTs were reviewed [29–37]. All of the trials were conducted in China and published in Chinese. The characteristics of the 14 randomized trials are shown in Table 1.

A total of 488 patients with AMS were included. Intervention included prescriptions of Chinese decoctions or traditional medicine monomers used alone or combined with Western drugs. The majority of included patients were young men aged between 13 and 42 years. Ten trials specified three diagnostic criteria of AMS, and only one trial [33] used the Lake Louise consensus on definition and quantification of altitude illness. Two trials [36, 37] used the Chinese Medical Association Third National Plateau Medicine of Academic Seminars (1996)—“The Naming, Classification and Diagnosis Standard of Altitude Sickness.” Six trials [29–32, 34, 35] used the Chinese State Military Standard GJB1098-91—“Principles of diagnosis and treatment of benign forms of acute mountain sickness.” Interventions included all of the Chinese prescriptions used alone or combined with western drugs. Chinese decoctions or traditional medicine monomers included Fufang yi hao pills, Sheng nao kang pills, Shu li kang capsules, Ginkgo leaf tablets, a new compound called rhodiola pills, Xing nao jing injection, Danhong injection, and the root of *Rhodiola rosae*. The controls included four types of groups, including Western drugs (acetazolamide), routine treatment, placebo, and no drugs. Seven trials investigated Chinese prescriptions used alone [29–35] versus control groups, and the remaining two trials [36, 37] compared Chinese prescriptions plus routine treatment drugs with routine treatment drugs. The treatment duration was in the range of 3–10 days. The various prescriptions are shown in Table 2. The different compositions of Chinese herbal formulas are also shown in Table 2. All of the nine trials used the score of AMS as the outcome measure. Adverse effects were described in detail. The main finding of the trials showed that the use of Chinese formulas or traditional medicine monomers had beneficial effects for the prevention and therapy of AMS.

### 3.2. Methodological Quality of Induced Trials

The majority of the included trials were assessed to have generally poor methodological quality according to the predefined quality assessment criteria (Table 3). The randomized allocation of participants was mentioned in all trials. However, only one trial stated the methods for sequence generation by a random number table [35]. Only one trial stated the double-blind principle [29]. Generally, insufficient information was provided to determine whether the trial was conducted properly. Allocation concealment and blinding of outcome assessment were not mentioned in all of the trials. None of the trials reported dropouts or withdrawals. None of the trials had a pretrial estimation of sample size.

### 3.3. Effect of Interventions

#### 3.3.1. Chinese Herbal Medicine versus Routine Western Drugs

Two trials [34, 35] compared prescriptions of Chinese formula used alone with Western drugs. A change in the score of AMS was reported in the two RCTs. Both of these trials showed homogeneity in the consistency of the trial results (chi-square = 4.76, *P* = 0.03; *I* ^2^ = 79%). Therefore, a random-effects model should have been used for statistical analysis. A meta-analysis showed a significant beneficial effect of Chinese formula used alone compared with Western drugs in decreasing the score of AMS (MD: −2.23 [−3.98, −0.49], *P* = 0.01).

#### 3.3.2. Chinese Herbal Medicine versus No Drug Therapy

One trial [33] compared prescriptions of Chinese formula used alone with no drugs. A change in the score of AMS was reported. This trial did not show homogeneity in the consistency of the trial results (chi-square = 7.58, *P* = 0.30; *I* ^2^ = 13%). Therefore, a fixed-effects model should have been used for statistical analysis. A meta-analysis showed a significant beneficial effect of Chinese formula used alone compared with no drug treatment in decreasing the score of AMS (MD: −6.00 [−6.45, −5.55], *P* < 0.00001).

#### 3.3.3. Chinese Herbal Medicine versus Placebo

Four trials [29–32] compared Chinese formula used alone with placebo. A change in the score of AMS was reported in all of the RCTs. The four trials did not show homogeneity in the consistency of the trial results (chi-square = 1.01, *P* = 0.80; *I* ^2^ = 0%). Therefore, a fixed-effects model should have been used for statistical analysis. A meta-analysis showed a significant beneficial effect of Chinese formula used alone compared with placebo treatment in decreasing the score of AMS (MD: −1.10 [−1.64, −0.55], *P* < 0.0001).

#### 3.3.4. Chinese Herbal Medicine Plus Routine Western Drugs versus Routine Western Drugs

Two trials [36, 37] compared the combination of Chinese formula plus routine treatment drugs with routine treatment drugs. A change in the score of AMS was reported in the two RCTs. Both of the trials showed homogeneity in the consistency of the trial results (chi-square = 235.19, *P* < 0.00001; *I* ^2^ = 82%). Therefore, a random-effects model should have been used for statistical analysis. A meta-analysis showed a significant beneficial effect of Chinese formula plus routine treatment drugs compared with routine treatment drugs in decreasing the score of AMS (MD: −5.99 [−11.11, −0.86], *P* = 0.02; Table 4).

### 3.4. Publication Bias

The number of trials was too small to conduct any sufficient additional analysis of publication bias.

### 3.5. Adverse Effects

Two of nine trials mentioned the presence or absence of adverse effects [29, 34]. One trial reported specific symptoms, such as mild diarrhea with Ginkgo leaf tablets. This adverse effect was not severe and it spontaneously recovered without special treatment. No adverse events were found in the other trial [34].

## 4. Discussion

Currently, the best method of preventing AMS is by ascending gradually and allowing time for acclimatization. If rapid ascent is inevitable, the carbonic anhydrase inhibitor acetazolamide is useful for prophylaxis. Acetazolamide produces metabolic acidosis by increasing renal excretion of bicarbonate, which in turn stimulates ventilation. The dosage of acetazolamide required is 250 mg once or twice daily, or 125 mg taken at night. However, side effects of acetazolamide are common and include diuresis, paresthesia of the fingers and toes, and a flat unpleasant taste to carbonated drinks. Dexamethasone is also effective in preventing acute mountain sickness, but there are side effects. Currently, alternative and synergistic treatment of TCM has drawn attention from scholars of different countries [38–41]. Research has shown that some Chinese herbal compounds and several traditional medicine monomers or Chinese herbal extracts effectively prevent AMS.

Based on our study and meta-analyses of the outcome on the score of AMS, Chinese herbal medicines may have a positive effect for prevention and therapy of AMS. Chinese herbal medicines as an adjunctive treatment significantly reduce the severity of AMS, alleviate symptoms of AMS, and reduce the incidence of AMS. Chinese herbal medicines are becoming more frequently used in China and Western countries. However, because of the unclear methodological quality of the trials included in our study, available data are not adequate to draw a definite conclusion of Chinese herbal medicines for AMS. Our positive findings should be interpreted conservatively.

Several limitations should be considered before accepting the findings of this study. First, formulations of Chinese herbal medicines in the nine included RCTs varied, such as single Chinese herbs, compound prescriptions, sterilized powder for injections, herbal decoctions, and herbal pills. Dosage and proportion were different for the same herb in different compound prescriptions. Additionally, there was variation in therapeutic principals of different research on the theory of TCM. Similar studies in a meta-analysis showed no statistical heterogeneity between them, but these factors may lead to potential clinical and methodological heterogeneity because of the differences existing in TCM formulas, duration of interventions, follow-up time, and measurement indicators.

Second, in terms of the current evaluative standards, the methodological quality of included studies was generally low. Bias may exist in many areas, such as an unclear method of random sequence, and no mention of allocation concealment and being double blind. There is also the possibility that bias was produced by the link of distribution, implementation and measurement, statistics, and reporting. However, our findings suggested that the overall effectiveness and cure rate of Chinese herbal medicine in the treatment of AMS were higher and better than Western medicine. Results of each RCT also suggested that the curative effect of Chinese herbal medicine was not lower than Western controlled drugs. However, it is difficult to make specific and reliable recommendations on treatment because of the shortage of methodology in the research itself and the evaluative standard.

Third, two of nine trials mentioned the presence or absence of adverse effects. One of these trials reported specific symptoms, such as mild diarrhea in the interventional group, but this was not severe. The diarrhea spontaneously recovered without special treatment. Another trial only mentioned that there were no adverse effects in their study. The other seven trials did not report any adverse effects and these were not significantly different between the two treatments. In China, it is widely believed that herbal medicines are safe for various diseases. With an increasing amount of reports of adverse effects of Chinese herbal medicines, the safety of Chinese herbs and formulae needs to be rigorously monitored in future clinical trials. Therefore, because of the limited and inadequate evidence provided by eligible trials, conclusions about the safety of Chinese herbal medicines cannot be made from this study. Large-scale clinical trials with long-term follow-up are warranted to properly assess the safety of new integrative medicine therapy.

Fourth, none of the included trials described the economic index. A recent investigation of 20 public hospitals and integrative medical hospitals in Beijing demonstrated that $4 million can be saved in medical expenses if prescriptions of Chinese herbal medicine were increased by 1% [42]. In view of this finding, the focus should be on health economics indices of Chinese herbal medicine treatment.

## 5. Conclusions

Because of the unclear methodological quality of trials included in this study, a definite conclusion on efficacy and safety associated with Chinese herbal medicine for AMS cannot be drawn. Before recommending Chinese herbal medicine as an alternative treatment measure in AMS patients, more rigorous high-quality trials are required to provide a high level of evidence.

## Figures and Tables

**Table 1:** Clinical trials of Chinese herbal interventions in treating AMS with a concomitant population.

Reference (year)	Study design	Participants T/C	Intervention (herbs included)	Control	Outcome measure	Treatment duration (days)
Chen et al. (2006) [29]	RCT	23/23	Fufang yi hao pill (3 pills, tid)	Placebo (3 pills, tid)	Score of AMS	7
Song et al. (2011) [30]	RCT	18/18	Sheng nao kang pill (10 pills, tid)	Placebo (10 pills, tid)	Score of AMS	10
Tang et al. (2013) [31]	RCT	26/15	Sheng nao kang pill (10 pills, tid)	Placebo (10 pills, tid)	Score of AMS	7
Niu et al. (2006) [32]	RCT	50/50	Shu li kang capsule (3 pills, bid)	Placebo (3 pills, bid)	Score of AMS	7
Li et al. (2008) [33]	RCT	9/19	Root of *Rhodiola rosea* (15 g, qd)	No drug	Score of AMS	7 d
Zhang et al. (2003) [34]	RCT	20/18	Ginkgo leaf tablet (2 pills, bid)	Acetazolamide (125 mg, bid)	Score of AMS	7
Zhang et al. (2010) [35]	RCT	30/15	New compound, rhodiola pill (2 pills, qd)	Acetazolamide (125 mg, bid)	Score of AMS	5
Fang (2008) [36]	RCT	40/38	Xing nao jing injection (20 mL, ivgtt, qd) plus routine treatment	Routine treatment	Score of AMS	3
Fang (2011) [37]	RCT	39/37	Danhong injection (20 mL, ivgtt) plus routine treatment	Routine treatment	Score of AMS	5

Bid: twice daily; tid: three times daily; qd: four times daily; T/C: treatment group and control group; CT: clinical trial; RCT: randomized clinical trial; NR: not reported.

**Table 2:** Ingredients of frequently used Chinese Formulas.

Formulas	Components	TCM efficacy
Fufang yi hao pill	Radix Ginseng [Ren shen, *人参*], Radix astragali [Huang qi, *黄芪*], Ginkgo [Yin xing, *银杏*].	Supplementing qi and promoting blood circulation for removing obstruction.

Sheng nao kang pill	Salvia miltiorrhiza [Dan shen, *丹参*], Panax notoginseng [San qi, *三七*], Rhizoma chuanxiong [Chuan xiong, *川芎*], Radix Paeoniae Rubra [Chi shao, *赤芍*], Radix astragali [Huang qi, *黄芪*], Angelica sinensis [Dang gui, *当归*], Codonopsis pilosula [Dang shen, *党参*], Hirudo [Shui zhi, *水蛭*], rhizoma gastrodiae [Tian ma, *天麻*], and so forth.	Promoting blood circulation to remove blood stasis, removing obstruction in the collaterals, cooling blood to decrease blood pressure, and relieving spasms by subduing liver wind.

Shu li kang capsule	Flos rosae rugosae [Mei gui hua, *玫瑰花*], Chinese wolfberry [Gou qi zi, *枸杞子*], *Rhodiola rosae* [Hong jing tian, *红景天*], Peach blossom [Tao hua, *桃花*], Radix astragali [Huang qi, *黄芪*], Semen Juglandis [He tao ren, *核桃仁*], Angelica sinensis [Dang gui, *当归*], and rhubarb [Da huang, *大黄*].	Regulating the circulation of qi and blood. Supplementing qi, nourishing blood, and promoting blood circulation for removing stasis.

New compound, rhodiola pill	Codonopsis pilosula [Dang shen, *党参*], Salvia miltiorrhiza [Dan shen, *丹参*], Angelica sinensis [Dang gui, *当归*], Radix glehniae [Bei sha shen, *北沙参*], and Radix tinosporae [Jin guo lan, *金果榄*].	Promoting blood circulation to remove blood stasis, supplementing qi, and tranquilizing the mind.

Danhong injection	Extract from Salvia miltiorrhiza [Dan shen, *丹参*] and safflower [Hong hua, *红花*].	Promoting blood circulation for removing blood stasis and removing obstruction in the collaterals.

Xing nao jing injection	Extract from musk [She xiang, *麝香*], Borneolum Syntheticum [Bing pian, *冰片*], Radix curcumae [Yu jin, *郁金*], and Fructus gardeniae [Zhi zi, *栀子*].	Eliminating heat and purging fire, cooling blood and detoxifying, and consciousness restoring resuscitation.

Ginkgo leaf tablet	Extract from leaf of Ginkgo [Yin xing, *银杏*].	Benefiting qi by activating blood circulation and removing obstruction in the collaterals.

*Rhodiola rosae* decoction	Root of *Rhodiola rosae* [Hong jing tian, *红景天*].	Strengthening the spleen and replenishing qi, clearing the lung to relieve coughing, and promoting blood circulation for removing blood stasis.

**Table 3:** Quality assessment of included randomized controlled trials.

Included trials	Random sequence generation	Allocation concealment	Blinding of participants and personnel	Blinding of outcome assessment	Incomplete outcome data	Selective reporting	Other sources of bias
Chen et al., 2006 [29]	Unclear	Unclear	Yes	Unclear	No	No	Unclear
Song et al., 2011 [30]	Unclear	Unclear	Unclear	Unclear	No	No	Unclear
Tang et al., 2013 [31]	Unclear	Unclear	Unclear	Unclear	No	No	Unclear
Niu et al., 2006 [32]	Unclear	Unclear	Unclear	Unclear	Unclear	No	Unclear
Li et al., 2008 [33]	Unclear	Unclear	Unclear	Unclear	No	No	Unclear
Zhang et al., 2003 [34]	Unclear	Unclear	Unclear	Unclear	No	No	Unclear
Zhang et al., 2010 [35]	Table of random numbers	Unclear	No	Unclear	No	No	Unclear
Fang, 2008 [36]	Unclear	Unclear	Unclear	Unclear	No	No	Unclear
Fang, 2011 [37]	Unclear	Unclear	Unclear	Unclear	No	No	Unclear

**Table 4:** Analysis of the score of AMS.

Trials		MD (95% CI)	*P* value
Chinese formula versus Western drugs			
Ginkgo leaf tablet versus acetazolamide	1	−1.20 [−2.69, 0.29]	0.14
A new compound, rhodiola pill, versus acetazolamide	1	−3.00 [−3.63, −2.37]	<0.00001

Meta-analysis	2	−2.23 [−3.98, −0.49]	0.01

Chinese formula versus no drugs			
Root of *Rhodiola rosae* versus no drugs	1	−6.00 [−6.45, −5.55]	<0.00001

Meta-analysis	1	−6.00 [−6.45, −5.55]	<0.00001

Chinese formula versus placebo			
Fufang yi hao pill versus placebo	1	−1.00 [−2.26, 0.26]	0.12
Sheng nao kang pill versus placebo	1	−1.67 [−3.24, −0.10]	0.04
Sheng nao kang pill versus placebo	1	−1.59 [−3.40, 0.22]	0.08
Shu li kang capsule versus placebo	1	−0.94 [−1.64, −0.24]	0.009

Meta-analysis	4	−1.10 [−1.64, −0.55]	<0.0001

Chinese formula plus routine treatment drugs versus routine treatment drugs			
Xing nao jing injection plus routine treatment drugs versus routine treatment drugs	1	−8.61 [−9.24, −7.98]	<0.00001
Danhong injection plus routine treatment drugs versus routine treatment drugs	1	−3.38 [−3.61, −3.15]	<0.00001

Meta-analysis	2	−5.99 [−11.11, −0.86]	0.02

## Abbreviations

AMS: Acute mountain sickness
CI: Confidence interval
RCT: Randomized controlled trial
TCM: Traditional Chinese medicine.
